# Increased Population Risk of *AIP*‐Related Acromegaly and Gigantism in Ireland

**DOI:** 10.1002/humu.23121

**Published:** 2016-10-04

**Authors:** Serban Radian, Yoan Diekmann, Plamena Gabrovska, Brendan Holland, Lisa Bradley, Helen Wallace, Karen Stals, Anna‐Marie Bussell, Karen McGurren, Martin Cuesta, Anthony W. Ryan, Maria Herincs, Laura C. Hernández‐Ramírez, Aidan Holland, Jade Samuels, Elena Daniela Aflorei, Sayka Barry, Judit Dénes, Ida Pernicova, Craig E. Stiles, Giampaolo Trivellin, Ronan McCloskey, Michal Ajzensztejn, Noina Abid, Scott A. Akker, Moises Mercado, Mark Cohen, Rajesh V. Thakker, Stephanie Baldeweg, Ariel Barkan, Madalina Musat, Miles Levy, Stephen M. Orme, Martina Unterländer, Joachim Burger, Ajith V. Kumar, Sian Ellard, Joseph McPartlin, Ross McManus, Gerard J. Linden, Brew Atkinson, David J. Balding, Amar Agha, Chris J. Thompson, Steven J. Hunter, Mark G. Thomas, Patrick J. Morrison, Márta Korbonits

**Affiliations:** ^1^ Centre of EndocrinologyWilliam Harvey Research Institute, Barts and the London School of Medicine and DentistryQueen Mary University of LondonLondonUK; ^2^ Department of EndocrinologyCarol Davila University of Medicine and PharmacyC.I. Parhon National Institute of EndocrinologyBucharestRomania; ^3^ Research Department of GeneticsEvolution and EnvironmentUniversity College LondonLondonUK; ^4^ Department of Medical GeneticsBelfast HSC TrustBelfastUK; ^5^ Regional Centre for Endocrinology and DiabetesRoyal Victoria HospitalBelfastUK; ^6^ Department of Molecular GeneticsRoyal Devon and Exeter NHS Foundation Trust/ Institute of Biomedical and Clinical ScienceUniversity of Exeter Medical SchoolExeterUK; ^7^ Department of Endocrinology and DiabetesBeaumont Hospital/RCSI Medical SchoolDublinIreland; ^8^ Department of Clinical Medicine and Institute of Molecular MedicineTrinity College Dublin, Trinity Centre for Health Sciences, St James's HospitalDublinIreland; ^9^ Royal Belfast Hospital for Sick ChildrenBelfastUK; ^10^ Endocrinology Service/Experimental Endocrinology UnitHospital de EspecialidadesCentro Medico Nacional Siglo XXIIMSSMexico CityMexico; ^11^ Department of Endocrinology and DiabetesBarnet General HospitalLondonUK; ^12^ Academic Endocrine UnitOCDEMUniversity of OxfordOxfordUK; ^13^ Department of Endocrinology and DiabetesUniversity College London HospitalsLondonUK; ^14^ Department of NeurosurgeryUniversity of MichiganAnn ArborMichiganUSA; ^15^ Department of EndocrinologyUniversity Hospitals of LeicesterLeicesterUK; ^16^ Department of EndocrinologySt James's University HospitalLeedsUK; ^17^ Institute of AnthropologyJohannes Gutenberg UniversityMainzGermany; ^18^ North East Thames Regional Genetics ServiceGreat Ormond Street HospitalLondonUK; ^19^ Trinity BiobankInstitute of Molecular MedicineTrinity College DublinTrinity Centre for Health SciencesSt James's HospitalDublinIreland; ^20^ Centre for Public HealthSchool of MedicineDentistry and Biomedical SciencesQueen's University BelfastBelfastUK; ^21^ School of BiosciencesUniversity of MelbourneParkvilleVictoriaAustralia; ^22^ Schools of Mathematics and StatisticsUniversity of MelbourneParkvilleVictoriaAustralia; ^23^ Centre for Cancer Research and Cell BiologyQueens University BelfastBelfastUK

**Keywords:** *AIP*, acromegaly and gigantism, evolutionary genetics, founder mutation, population screening

## Abstract

The aryl hydrocarbon receptor interacting protein (*AIP*) founder mutation R304^*^ (or p.R304^*^; NM_003977.3:c.910C>T, p.Arg304Ter) identified in Northern Ireland (NI) predisposes to acromegaly/gigantism; its population health impact remains unexplored. We measured R304^*^ carrier frequency in 936 Mid Ulster, 1,000 Greater Belfast (both in NI) and 2,094 Republic of Ireland (ROI) volunteers and in 116 NI or ROI acromegaly/gigantism patients. Carrier frequencies were 0.0064 in Mid Ulster (95%CI = 0.0027–0.013; *P* = 0.0005 vs. ROI), 0.001 in Greater Belfast (0.00011–0.0047) and zero in ROI (0–0.0014). R304^*^ prevalence was elevated in acromegaly/gigantism patients in NI (11/87, 12.6%, *P* < 0.05), but not in ROI (2/29, 6.8%) versus non‐Irish patients (0–2.41%). Haploblock conservation supported a common ancestor for all the 18 identified Irish pedigrees (81 carriers, 30 affected). Time to most recent common ancestor (tMRCA) was 2550 (1,275–5,000) years. tMRCA‐based simulations predicted 432 (90–5,175) current carriers, including 86 affected (18–1,035) for 20% penetrance. In conclusion, R304^*^ is frequent in Mid Ulster, resulting in numerous acromegaly/gigantism cases. tMRCA is consistent with historical/folklore accounts of Irish giants. Forward simulations predict many undetected carriers; geographically targeted population screening improves asymptomatic carrier identification, complementing clinical testing of patients/relatives. We generated disease awareness locally, necessary for early diagnosis and improved outcomes of *AIP*‐related disease.

## Introduction

Germline aryl hydrocarbon receptor interacting‐protein (*AIP*; MIM# 605555) mutations cause autosomal dominant familial isolated pituitary adenomas (FIPA) most commonly manifesting as acromegaly or gigantism. Due to incomplete penetrance, the disease can also manifest as apparently sporadic pituitary adenoma (PA) [Vierimaa et al., 2006; Daly et al., 2007; Leontiou et al., 2008]. These mostly GH‐(somatotrophinomas) and/or prolactin‐secreting tumors develop in 17%–20% of *AIP* mutation (*AIP*mut) carriers, typically before 30 years of age [Beckers et al., 2013; Hernández‐Ramírez et al., 2015]. A diagnostic delay of several years from onset of symptoms is typical in somatotrophinomas, allowing tumor expansion and prolonged GH excess, leading to significant complications [Reddy et al., 2010]. Despite major therapeutical advances, management of somatotrophinomas remains challenging, especially in young patients with large/invasive tumors, two frequent characteristics of *AIP*mut‐positive patients [Beckers et al., 2013]. Identification of *AIP*mut carriers enables earlier clinical diagnosis and treatment, improving outcomes [Hernández‐Ramírez et al., 2015]. To this end, predictive genetic testing of relatives of carriers is recommended [Korbonits et al., 2012]. General population screening is costly but targeted screening could be cost effective when a disease‐associated allele is geographically highly localized [Schiavi et al., 2012], as might be expected for evolutionarily recent mutations. To date, population screening for genetic pituitary disease has not been performed.

We have previously inferred a recent origin for the NM_003977.3:c.910C>T (p.Arg304Ter) allele (p.R304^*^ or R304^*^) in six FIPA pedigrees of Irish ancestry, including individuals born in the 18th and 20th centuries, in a small region within Mid Ulster, Northern Ireland (NI) [Chahal et al., 2011; Stals et al., 2011]. Using the age estimate of R304^*^, we predicted through forward simulations a large number of current carriers [Chahal et al., 2011]. Therefore, we hypothesized that R304^*^ would be frequent in the Irish general population, in particular in Mid Ulster, and should lead to a high proportion of R304^*^‐positive patients with somatotrophinomas. In this study, we conducted a population screening in Mid Ulster, in comparison with two large population samples from Greater Belfast, NI, and from the Republic of Ireland (ROI). In addition, we identified R304^*^ carriers among patients with acromegaly/gigantism in two main Irish endocrine referral centers (Belfast and Dublin) and studied R304^*^‐positive patients in our International FIPA Consortium database [Hernández‐Ramírez et al., 2015]. We developed a new coalescent‐based simulation approach to more precisely estimate the time to most recent common ancestor (tMRCA) of the Irish R304^*^ allele based on haplotype data, and applied forward simulation to predict the number of carriers currently alive.

## Materials and Methods

### Subjects

We performed a cross‐sectional genetic analysis study of general population adult individuals from three regions in Ireland: screening of volunteers from Mid Ulster (Local Government Districts of Magherafelt, Cookstown, and Dungannon of NI) and genotyping of two large control groups from the Greater Belfast region—PRIME study participants [Linden et al., 2012]—and the ROI Trinity Biobank (Table 1). Sample size calculations and screening procedures are described in the Supp. Methods.

**Table 1:** Demographic Parameters of Irish Population Samples

	Mid Ulster (*n* = 936)	Greater Belfast (*n* = 1,000)	ROI (*n* = 2,094)	
Sample type	Saliva DNA	Blood DNA	Blood DNA (*n* = 1,095)	Buccal swab DNA (*n* = 999)
Males, count (proportion)	435 (46.5%)	1,000 (100%)	486 (44.4%)	559 (55.9%)
Age, years, median (range)	46 (18–85)	64 (58–72)	37 (19–69)	53 (18–76)
R304^*^/wt *AIP* genotype	6	1^a^	0	0
wt/wt *AIP* genotype	930	999	1,095	999
R304^*^ carrier frequency	6/936	1/1,000	0	0

a Seventy one years old male, normal height, no clinical signs of pituitary disease, normal serum hormone levels (basal IGF–I, prolactin, and GH serum levels during the oral glucose tolerance test (GH–OGTT)), normal pituitary MRI examination—subsequently died of a pulmonary mesothelioma linked to asbestos exposure, aged 72 years; cascade testing of relatives (Screening 2 pedigree) identified two unaffected R304^*^ carriers: one male, 77 years, clinically unaffected and one female, 42 years, clinically unaffected, normal pituitary MRI, random GH = 4.7 ng/ml, minimal IGF–I increase (1·07 × ULN), normal PRL, GH–OGTT not yet performed. wt, wild–type

A prospective cohort of unselected patients with acromegaly or gigantism were recruited at the University Hospital in Belfast, the only tertiary pituitary referral center in NI (*n* = 87, representing 60% of the NI Acromegaly Registry patients), and at the Beaumont Hospital, Dublin, the largest pituitary referral center in ROI and closest to Mid Ulster (*n* = 29). Additional R304^*^‐positive pedigrees (five Irish and five non‐Irish) and 13 *AIP*mut‐negative Irish FIPA pedigrees [Hernández‐Ramírez et al., 2015] were also included. Ethnicity—as assessed through family history—was unambiguous for all pedigrees. R304^*^‐positive patients were previously reported [Leontiou et al., 2008; Igreja et al., 2010; Chahal et al., 2011; Stals et al., 2011; Williams et al., 2014; Hernández‐Ramírez et al., 2015], while the other patients and subjects have not been previously described. Patients were tested for *AIP*muts, as previously described [Igreja et al., 2010]. The following pedigree codes were used: “Sp” (sporadic), “FIPA” (familial) and “Screening” (screening detected). Tooth‐extracted DNA from a pituitary giant's skeleton at Trinity College Dublin [Cunningham, 1892] was sequenced for *AIP*mut, as previously described [Chahal et al., 2011]. The study protocol was approved by the local Ethics Committees; all study participants gave written informed consent.

### Evolutionary Genetics and Statistical Analysis

Fourteen microsatellites (STR) covering 8.3 Mbp around the *AIP* gene were genotyped in a minimum of one R304^*^ carrier per pedigree, as previously described [Chahal et al., 2011]. Haplotypes were computed using PHASE [Stephens et al., 2001], incorporating phasing information deduced from genotypes of closely related carriers (three pedigrees) (Supp. Table S1). To estimate the tMRCA of R304^*^‐containing haplotypes, we developed a novel inference methodology combining an analytical result from coalescence theory and simulations in an Approximate Bayesian Computation framework [Beaumont et al., 2002]. First, we computed the tMRCA based on a smaller haplotype block around R304^*^ that was fully conserved in 18 Irish pedigrees (Supp. Table S2, row E). The full conservation allowed us to apply an analytical formula [Donnelly et al., 1996]. The tMRCA distribution resulting from this first step became the prior distribution for the second step that used a simulation‐based approach to incorporate additional conservation data of haploblocks shared only between subsets of pedigrees. We simulated genealogies with recombination and mutation using the program *ms* [Hudson, 2002] (Supp. Fig. S1) and generated distributions of tMRCA from those simulations that were closest to the recombination and mutation pattern inferred from our haplotype data (Supp. Fig. S2). Results were based on sex‐averaged genetic distances for *AIP* and microsatellite loci, from HapMap v2 [The International HapMap Consortium, 2007] and Rutgers v3 [Matise et al., 2007] genetic maps (Supp. Table S1). Next, starting with tMRCAs randomly chosen from the simulated tMRCA distribution (Supp. Fig. S3A and B), we took a forward simulation approach to generate possible trajectories for the allele frequency in the population, through binomial sampling. This resulted in distributions of the expected present‐day number of carriers, which we conditioned to have at least the minimum number of R304^*^ carriers/generation observed in our dataset (*n* = 27) (Supp. Fig. S3C and D). Detailed procedures, including statistical analysis are described as Supp. Methods.

## Results

### Population Screening Reveals a Significantly High Carrier Frequency of the R304^*^ Allele in NI

Several families of patients suffering from acromegaly or gigantism and carrying the Irish R304^*^ allele came from a small geographical area within Mid Ulster in NI [Chahal et al., 2011; Stals et al., 2011]. In order to estimate the local carrier frequency of R304^*^, we screened a population sample of 936 adults (Table 1), 90% of which lived in Mid Ulster.

We identified six R304^*^ heterozygous individuals (Supp. Table S3), corresponding to a carrier frequency estimate of 0.0064 (95% probability interval [CI]: 0.0027–0.013). Two related carriers, negative for personal or family history of PA, represented a novel R304^*^ pedigree (Screening 1) (Supp. Tables S3 and S4). The remaining carriers detected through screening were two patients previously diagnosed with somatotrophinomas (pedigrees Sp 4 and FIPA 4) and two unaffected carriers from previously identified R304^*^‐positive pedigrees Sp 4 and FIPA 1 (Supp. Tables S3 and S4). The R304^*^ carrier status was significantly associated with a personal diagnosis of PA (*P* < 0.001, Fisher's exact test) and with a family history of PA (FIPA or sporadic, *P* < 0.001, Fisher's exact test). Mid Ulster screening revealed many other PA patients, either directly (R304^*^‐negative PA patients participating to screening) or indirectly (nonparticipating PA patients mentioned as family history of R304^*^‐negative screening subjects) (Supp. Table S3).

The Greater Belfast control population sample (*n* = 1,000, Table 1) revealed one R304^*^‐positive individual; corresponding to a carrier frequency of 0.001 (95% CI: 0.00011–0.0047). The carrier was unaffected (clinical examination, hormonal measurements, and pituitary imaging), did not have a family history of PA, and cascade testing of relatives revealed two carriers, both unaffected (Supp. Table S4, Screening 2 pedigree).

In the ROI control population sample (*n* = 2,094, Table 1), no R304^*^‐positive individuals were identified, corresponding to zero carrier frequency (95% CI estimate 0–0.0014). This is in accord with the absence/extremely low R304^*^ carrier frequency observed in diverse population samples included in the Exome Aggregation Consortium (http://exac.broadinstitute.org/): two heterozygotes in 34,856 Europeans (0.000057) and none in 23,450 non‐Europeans. The Mid Ulster carrier frequency was significantly higher than the ROI one (*P* = 0.0005, Fisher's exact test), while the Greater Belfast one did not differ from either ROI or Mid Ulster.

Extrapolation of our NI R304^*^ carrier frequency estimates (1/1,000 and 6/936) and 95% confidence intervals (CIs) to the population of Greater Belfast (579,276) and Mid Ulster (139,011), comprising 40% of NI according to the 2011 census (http://www.ninis2.nisra.gov.uk), yielded an estimated number of carriers of 1,470 (95% CI: 439–4,530).

### R304^*^ is Common in Irish Patients with Acromegaly/Gigantism

In order to characterize the extent of R304^*^‐related pituitary disease in Ireland, we tested for *AIP* alleles in 116 patients with acromegaly/gigantism from the two tertiary endocrine centers close to Mid Ulster. We identified 11 (12.6%) R304^*^‐positive patients with somatotrophinomas in the NI Acromegaly Registry, Belfast, and two (6.8%) in the Dublin center patients, while five NI (5.7%) and two Dublin patients (6.8%) presented other *AIP* variants (Supp. Table S5).

**Figure 1:**
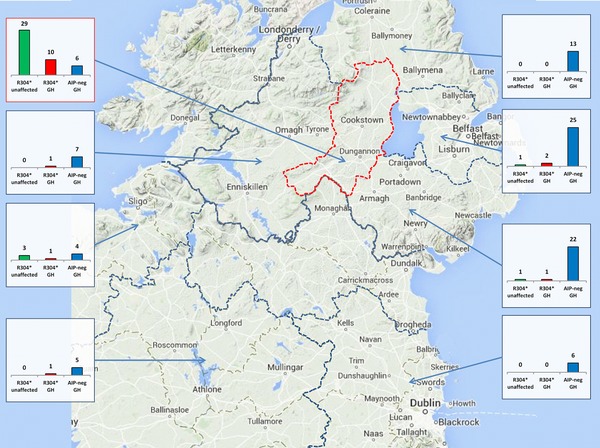
Geographical distribution of R304^*^ allele carriers in Ireland, compared to *AIP*mut–negative patients with somatotrophinomas. R304^*^ carriers, unaffected (R304^*^ unaffected, green columns) and patients with somatotrophinomas (R304^*^ GH, red columns) were compared to *AIP*mut–negative patients with somatotrophinomas (*AIP*–neg GH, blue columns). Only subjects currently residing in Ireland were included; R304^*^–positive patients with other PA types (*n* = 5) are not shown. The map was arbitrarily divided into regions separated by dotted borders, red for Mid Ulster. Ninety percent of the Mid Ulster screening subjects resided here, including all six R304^*^ carriers. Each small graph summarizes data from the region indicated by the corresponding arrow; the y–axis scale is identical for all graphs.

The R304^*^‐positive patients belonged to pedigrees FIPA 1–5 and Sp 1–6 (Supp. Table S4). The R304^*^ prevalence among NI patients with somatotrophinomas was higher than that of large non‐Irish somatotrophinoma series [Tichomirowa et al., 2011; Cazabat et al., 2012; Preda et al., 2014; Schöfl et al., 2014] (0–2.41%; *P* < 0.05, Fisher's exact test). Geographically, R304^*^‐positive patients with somatotrophinomas predominated in Mid Ulster, where they, remarkably, outnumbered *AIP*mut‐negative patients (Fig. 1).

Irish R304^*^‐positive patients with somatotrophinomas (excluding those diagnosed prospectively, following predictive genetic testing) displayed the known features of *AIP*mut‐positive PAs: younger age at disease onset/diagnosis, larger pituitary tumors, increased frequency of gigantism and familial disease, compared to *AIP*mut‐negative patients (Table 2).

**Table 2:** Clinical Characteristics of Irish R304^*^–Positive Somatotrophinoma Patients (NI Acromegaly Registry, Dublin patients and FIPA Consortium database) Compared to *AIP*mut–Negative Irish Patients with Somatotrophinomas (NI and Dublin Patients)

*AIP* genotype	Heterozygous NM_003977.3:c.910C>T *n* = 31	Homozygous wild‐type *n* = 96	*P* value
Median age (range) at onset (years)	17 (6–68) (*n* = 29)	38.5 (14–85) (*n* = 64)	<0.0001
Median age (range) at diagnosis (years)	19.5 (7–68) (*n* = 30)	46 (19–85) (*n* = 95)	<0.0001
Age <30 years at disease onset	27/31 (87.1%)	21/96 (21.9 %)	<0.0001
Mean tumor size (mm)	23.8 ± 10.6 (*n* = 11)	17.2 ± 8.4 (*n* = 43)	<0.05
Pituitary macroadenoma	22/24 (91.7%)	58/70 (82.9%)	ns
Gigantism	18/31 (58.1%)	7/96 (7.3%)	<0.0001
Family history of PA	24/31 (77.4%)	3/94 (3.2%)	<0.0001

ns = not significant

Our investigations revealed, however, a wider range of phenotypes. While two patients, diagnosed as part of cascade testing in families, harbored macroadenomas and underwent surgery, two prospectively diagnosed R304^*^‐positive patients had pituitary microadenomas and four patients had disease onset after the age of 30 years [Hernández‐Ramírez et al., 2015]. Interestingly, none of the patients carrying other *AIP* variants presented a PA family history.

### All Irish R304^*^ Pedigrees Share a Common Ancestor That Lived Approximately 2,500 Years Ago

**Figure 2:**
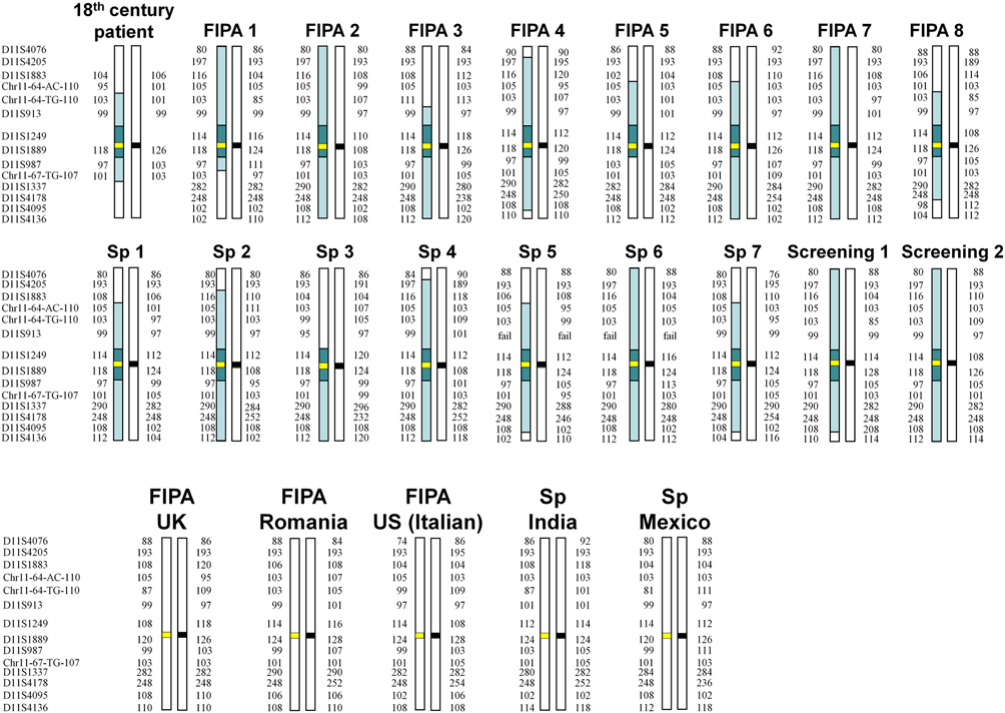
Microsatellite haplotypes of chromosome 11q12.2–13.3 of R304^*^–positive pedigrees. Irish (first two rows) and non–Irish pedigrees (third row) are shown. Marker alleles are displayed as amplicon sizes. Dark shading: haploblock shared between all Irish pedigrees (0.2–1.95 Mbp long); light shading: additional shared haploblocks. Thick horizontal lines represent *AIP* alleles (black = wild–type, yellow = R304^*^); intervals between markers are not drawn to scale. Sp, sporadic PA. Haplotypes of 18th century patient, FIPA 1, 2, 3, 6, 7, FIPA UK, Romania, US (Italian), Sp India and Sp Mexico pedigrees have been previously published (Chahal et al., 2011; Stals et al., 2011; Ramirez‐Renteria et al., 2016).

Having identified 13 R304^*^‐positive pedigrees through population screening and testing of somatotrophinoma patients in Ireland, we searched the International FIPA Consortium database for additional R304^*^‐positive pedigrees and identified 10 more, resulting in a total of 23 R304^*^‐positive pedigrees (Fig. 2). These 10 additional pedigrees were of diverse ethnic backgrounds: six from the UK: one of English and five of Irish ancestry (FIPA 6–8, Sp 7 and 18th century patient pedigrees) and four from other countries (Fig. 2 and Supp. Table S4). In this database, we also identified 13 *AIP*mut‐negative Irish FIPA pedigrees (Supp. Table S6).

By reconstructing *AIP* gene region STR haplotypes in these 23 pedigrees, we determined that 18 of them shared a 0.2–1.95 Mbp fully conserved haploblock around R304^*^, as well as extended haploblocks covering up to the entire 8.3 Mbp genotyped region (Fig. 2 and Supp. Table S1). These data support the existence of a recent common ancestral carrier haplotype, the Irish R304^*^ founder. Fourteen of these 18 pedigrees were living in Ireland and the other four had known Irish ancestry. The five non‐Irish pedigrees did not share significant haploblocks with the Irish ones or among each other (Fig. 2), supporting the existence of recurrent independent R304^*^ mutation events at this mutation‐prone CpG dinucleotide [Vierimaa et al., 2006; Daly et al., 2007; Leontiou et al., 2008; Occhi et al., 2010; Chahal et al., 2011; Tichomirowa et al., 2011; Cazabat et al., 2012; de Lima et al., 2012; Cuny et al., 2013; Niyazoglu et al., 2014].

The total number of Irish allele carriers was 90 (37 affected, 53 unaffected), 81 of which are currently alive (30 affected, 51 unaffected). Seven of the 51 unaffected living carriers (13.7%) were detected exclusively by population screening. The highest geographical concentration of R304^*^ carriers in Ireland was observed in Mid Ulster, both for affected (somatotrophinoma) and unaffected individuals (Fig. 1), corresponding to eight different pedigrees. A third of the Irish allele carriers (32%, 26/81) lived outside of Ireland: in England (21), Scotland (2), Australia (2), and Canada (1).

To estimate the time to the most recent common ancestor (tMRCA) of the R304^*^ carrier haplotypes among the Irish pedigrees, we developed a novel coalescent‐based approach, combining analytical calculations with haplotype simulation in an approximate Bayesian computation [Beaumont et al., 2002] framework (Supp. Methods). The tMRCA estimates of the Irish R304^*^ haplotypes were 102 (51–200) generations—median (95% CI)—based on HapMap and 101 (50–179) generations based on Rutgers map genetic distances (Supp. Fig. S3A and B). These tMRCA distributions, for example, 51–200 generations (HapMap‐based) overlap substantially with those previously estimated: 17–150 generations [Chahal et al., 2011] (Pr(X>Y) = 0.79). Assuming a generation time of 25 years [Chahal et al., 2011], current estimates translate to 2,550 (1,275‐5,000) years (HapMap) and 2,525 (1,250‐4,475) years (Rutgers). To estimate the number of living subjects carrying the Irish R304^*^ allele, forward simulations conditioned on the number of observed carriers predicted 144 (30–1,725) and 141 (30–1,430) carriers per generation—median (95% CI)—based on HapMap and Rutgers distances, respectively (Supp. Fig. S3C and D). For three overlapping generations alive at present, we estimate the current number of carriers as 3 × 144 = 432 (90‐5,175; HapMap‐based), corresponding to 86 (18–1,035) PA patients, assuming 20% penetrance [Beckers et al., 2013].

### Historical Irish Giants

**Figure 3:**
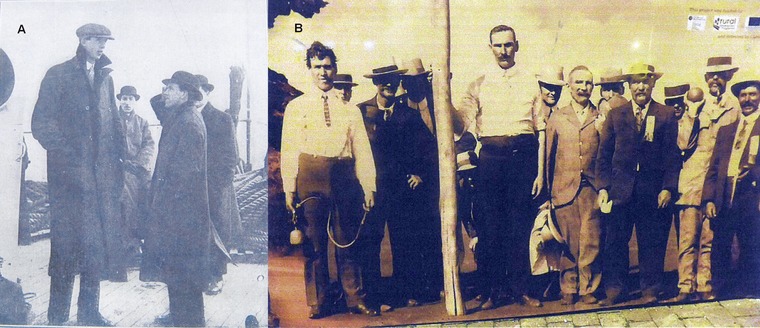
New evidence of historical Irish giants. (**A**) Subject 15 (Supp. Table S7), 211cm tall, from Maghera, Mid Ulster, uncle of a screening participant, died of a hypertensive crisis one month after photograph was taken – 13^th^ May 1918, Daily Sketch (Canadian newspaper). (**B**) Group of Irish immigrants from Garvagh (a town close to Mid Ulster) after arrival in Western Australia, 1910. At center with the pole, Subject 16 (Supp. Table S7), 213 cm tall; standing far left—unrelated subject with pronounced acromegalic features and tall stature (photograph from Garvagh Museum, NI).

The medical literature, as well as Irish folklore, holds numerous descriptions of Irish giants. For some of these presumed pituitary gigantism individuals there is historical and medical evidence for the diagnosis (Supp. Table S7). We have tested DNA samples from two historical giants for *AIP*‐derived alleles: Charles Byrne (1,761–1,783), born in Mid Ulster, who carried the Irish R304^*^ allele [Chahal et al., 2011] and Cornelius Magrath (1,736–1,760), born in the Southern coast of Ireland and whose skeleton is conserved at Trinity College, Dublin [Cunningham, 1892], who was R304^*^‐negative. Five additional giants related to known R304^*^ carriers were presumed R304^*^‐positive: Subjects 6, 7, 11, 13, and 19 (Supp. Table S7). Two unrelated Mid Ulster screening participants, R304^*^‐negative themselves, provided photographic evidence of their extremely tall relatives, whom we included as historical Irish giants (Fig. 3 and Supp. Table S7).

## Discussion

In this study we demonstrated that the R304^*^ *AIP* allele is present in the general population of Ireland and its frequency varies geographically, from a high of 6/936 in Mid Ulster to 1/1,000 in the Greater Belfast region (both in NI), while absent in a ROI population sample. These differences were paralleled by the geographical distribution of R304^*^‐positive somatotrophinoma cases, representing a significantly higher proportion of the somatotrophinoma patient population in NI (12.6%), than in large published somatotrophinoma cohorts (maximum 2.41%) [Tichomirowa et al., 2011; Cazabat et al., 2012; Preda et al., 2014; Schöfl et al., 2014], while in the ROI (Dublin center) this proportion was intermediate (6.8%). Furthermore, the number of R304^*^ carriers, both patients with somatotrophinomas and unaffected carriers, was highest in Mid Ulster. These data strongly support our hypothesis that Mid Ulster has an elevated R304^*^ carrier frequency. However, 32% of the Irish R304^*^ allele carriers were identified outside of Ireland, an observation attributable to emigration. Another consequence of the increased R304^*^ carrier frequency in Ireland is that a large proportion (8 of 21, 38%) of Irish FIPA families are due to R304^*^—the only *AIP*mut identified in Irish FIPA so far.

We demonstrate that all 18 R304^*^‐positive pedigrees of Irish origin identified in the study inherited this allele from a common ancestor, the R304^*^founder. In addition, we have shown that R304^*^ in pedigrees of English, Indian, Romanian, US Italian, Irish, and Mexican origin derives from independent recurrent mutational events [Chahal et al., 2011; Ramirez‐Renteria et al., 2016]. Founder *AIP*mut alleles have also been identified through haplotype analysis in Finnish [Vierimaa et al., 2006], Italian [Occhi et al., 2010], Comoros [Cuny et al., 2013], and English [Salvatori et al., 2014] populations, involving the p.Q14^*^, p.R304^*^, p.G117Afs^*^39, and p.F269_H275dup alleles, respectively, but the present population frequency of all these founder alleles remains unknown.

The refined coalescent simulation‐based approximate Bayesian computation approach we describe in this article provides a practical framework applicable to the estimation of tMRCA of identity‐by‐descent alleles. Previous estimates using five pedigrees and a simpler approach indicated a more recent common ancestor (66 generations, 95% CI = 17–150) [Chahal et al., 2011], although the difference between new and previous estimates was not statistically significant (Pr(X>Y) = 0.79). Although the founder probably lived around 2,500 years ago, many of today's carriers are concentrated in a small region, suggesting a limited local migratory activity, despite significant emigration in recent centuries. A population bottleneck followed by reexpansion may explain the Mid Ulster R304^*^ cluster, although genetic drift in a constant sized or growing population is consistent with observed frequencies and estimated age of this allele. Persistence of the R304^*^ allele in this population may suggest the absence of, or only weak, purifying selection acting on this locus. While R304^*^‐associated disease manifestations include hypopituitarism, infertility, and shortened life span, low disease penetrance will lead to a reduced selective disadvantage. Additionally, it is possible that there were inclusive fitness effects in the unaffected carrier relatives of giants in the past—perhaps as a result of elevated status—that may have counterbalanced selective disadvantages in affected carriers.

Identification of asymptomatic *AIP*mut carriers enables prospective early diagnosis of PAs, followed by prompt and effective treatment, with improved chances of curing the disease and preventing complications [Williams et al., 2014; Hernández‐Ramírez et al., 2015]. To this end, cascade testing of *AIP*mut‐positive patient relatives is recommended; however, limited knowledge of family relations, nondisclosure of information between relatives and nonacceptance of testing may hinder these efforts. While carrier identification in affected individuals is now becoming standard, detection of unaffected carriers and their endocrine screening is a challenge for the future. Supported by substantial patient and community involvement, and by local media coverage, our study has created awareness about *AIP*‐related disease in Ireland, a necessary precursor for improved disease recognition and acceptance of genetic testing. Although population screening is an unbiased approach to carrier identification and could detect unaffected carriers without family history of pituitary disease, further studies are needed to justify this approach. Our study provides a proof‐of‐principle that asymptomatic R304^*^ carriers in Ireland can be identified through population screening, by identifying two novel R304^*^‐positive pedigrees comprising a relatively large proportion (7/51, 13.7%) of the known number of living unaffected carriers.

Given the tMRCA‐predicted number of Irish R304^*^ carriers (432, 95% CI: 90–5,175, 86 affected expected at 20% penetrance) and that of known living carriers (81, of which 30 affected), we estimate the number of undetected carriers as the difference between predicted and known carriers, resulting in around 351 undetected carriers, including 56 affected patients. Many of these undetected carriers may actually live outside of Ireland, in countries with significant Irish immigration, such as Great Britain, Canada, and Australia, where 32% of the known living Irish allele carriers reside, and the USA, where *AIP* mutation testing has been limited to date. For NI, alternative calculations by extrapolating population carrier frequency estimates (Mid Ulster and Greater Belfast) to regional population size (approximately 40% of NI), estimated 340 to 5,138 undetected carriers, a range comparable to the tMRCA‐predicted estimate.

Several key features contribute to our study's results and impact: (1) patient and general public involvement in its design and conduct, (2) analysis of multiple Irish general population samples and of a large unbiased patient cohort—the NI Acromegaly Registry, (3) analysis of the largest R304^*^ pedigree collection to date, thanks to international collaboration, and (4) the incorporation of historical and ancient DNA data into our analyses. In comparison, studies describing other *AIP*mut founder alleles, as well as our own previous study of the Irish R304^*^ allele, examined limited number of *AIP*mut pedigrees and relatively small number of patients and population controls, without providing estimates of population carrier frequencies of these alleles and their geographical distribution [Vierimaa et al., 2006; Occhi et al., 2010; Chahal et al., 2011; Cuny et al., 2013; Salvatori et al., 2014].

Our study also has limitations. For practical reasons, only three large Irish general population samples were screened for R304^*^. Regarding PA types included in the study, we chose to include only patients with somatotrophinomas, as this is the commonest PA type in *AIP*mut carriers and the only one for which population‐level data were available. Patients with prolactinomas can also harbor *AIP* mutations, although significantly less frequently than somatotrophinomas [Tichomirowa et al., 2011; Cazabat et al., 2012; Cuny et al., 2013; Preda et al., 2014]; the frequency of R304^*^ in Irish patients with prolactinomas remains to be determined. Sampling of patients with somatotrophinomas from ROI was limited to the Dublin center, based on its proximity to Mid Ulster, and fewer patients were analyzed, compared to NI. Future studies will help to better characterize the R304^*^ carrier frequency in the ROI general and PA patient populations. Not all living members of R304^*^‐positive pedigrees were available for genetic and clinical testing; this is a common difficulty in genetic studies and clinical practice. Our evolutionary genetics approach compensated for this, by estimating the number of undetected allele carriers. Overall, we believe that these limitations have relatively little effect on our conclusions.

Our work suggested several measures to address the challenging task of identifying the large number of undetected Irish R304^*^ allele carriers, in order to improve the outcomes of PA patients. (1) We propose R304^*^ testing of patients of Irish descent with somatotrophinomas, especially ones with younger (<30 years of age) onset, in Ireland and abroad, particularly in Great Britain, USA, Canada, and Australia, where large Irish immigrant populations exist. (2) Working closely together with patients and their communities has improved knowledge about *AIP*‐related disease, which may lead to earlier recognition of disease, and hopefully increased acceptance of genetic testing. (3) Population screening in Ireland, especially in Mid Ulster, might be effective, but more study is needed regarding the psychological and economic implications, as well as further data on the clinical benefits of R304^*^ screening [Hernández‐Ramírez et al., 2015].

Our results open the way for future studies of several key aspects of *AIP*‐related disease. The Irish R304^*^ cohort is unique in having a large number of carriers sharing the same pathogenic gene alteration and promoter area within the fully conserved haploblock, yet pedigrees display differences in penetrance and patient phenotypes differ significantly. Currently, it is not known what influences disease penetrance in *AIP*mut‐positive individuals. Different individual *AIP*mut alleles [Igreja et al., 2010] or classes (e.g., truncating vs. nontruncating) [Hernández‐Ramírez et al., 2015], changes in gene expression regulation [Cooper et al., 2013], and other epistatic interactions may each play a role, as will other genetic and environmental factors. Further genetic studies of this cohort could help the identification of pituitary disease‐modifying genes. Follow‐up of clinically‐identified and screening‐detected pedigrees will provide new data about the natural history of disease in carriers, optimal follow‐up schedules, efficiency and outcome of population screening.

Irish folklore has numerous stories regarding Irish giants and the remains of some of these giants have been studied in the past [Cunningham, 1892; Frankcom and Musgrave, 1976; Chahal et al., 2011]. Our data provides an explanation for the observation made by the pioneering anthropologist James C. Prichard in 1826: In Ireland men of uncommon stature are often seen, and even a gigantic form and stature occur there much more frequently than in this island [Britain]… We can hardly avoid the conclusion that there must be some peculiarity in Ireland which gives rise to these phenomena [Prichard, 1826].

*Disclosure statement*: M.K. received grant funding from Novartis and Pfizer and is a member of Medical Advisory Board of Pfizer Inc. All the other authors have nothing to declare.

## Supporting information

Disclaimer: Supplementary materials have been peer‐reviewed but not copyedited.

Supp. Methods, Supp. Figures S1‐S3, Supp. Tables S1‐S7 and references [Austerlitz et al., 2003; Bergland, 1965; Burger et al., 2007; Carleton 1996; Colombo, 2007; Fawcett, 1909; Frankcom and Musgrave 1976; Georgitsi et al., 2007; Lin et al., 2007; Lynass 1842; Oriola et al., 2013; Prezio et al., 1961; Raitila et al., 2010; Stephens et al., 1998; Templeton 2006; Trivellin et al., 2014] can be found in the online version of this article under “Supporting Information”.

Supp. Methods, Supp. Figures S1‐S3, Supp. Tables S1‐S7 and references [Austerlitz et al., 2003; Bergland, 1965; Burger et al., 2007; Carleton 1996; Colombo, 2007; Fawcett, 1909; Frankcom and Musgrave 1976; Georgitsi et al., 2007; Lin et al., 2007; Lynass 1842; Oriola et al., 2013; Prezio et al., 1961; Raitila et al., 2010; Stephens et al., 1998; Templeton 2006; Trivellin et al., 2014] can be found in the online version of this article under “Supporting Information”.
