# Supplementary material for: Increased Population Risk of AIP‐Related Acromegaly and Gigantism in Ireland
Source: Hum Mutat. 2016 Oct 4;38(1):78–85. doi: 10.1002/humu.23121 (PMC5215436; doi:10.1002/humu.23121)
Supplement: Supplementary file 2 — Supp. Methods, Supp. Figures S1‐S3, Supp. Tables S1‐S7 and references [Austerlitz et al., 2003; Bergland, 1965; Burger et al., 2007; Carleton 1996; Colombo, 2007; Fawcett, 1909; Frankcom and Musgrave 1976; Georgitsi et al., 2007; Lin et al., 2007; Lynass 1842; Oriola et al., 2013; Prezio et al., 1961; Raitila et al., 2010; Stephens et al., 1998; Templeton 2006; Trivellin et al., 2014] can be found in the online version of this article under “Supporting Information”. [file HUMU-38-78-s002.pdf]

Supporting Information

Contents

Supplementary Methods 1

Sample size calculation 1

Mid Ulster screening and genotyping of population control groups 2

Gigantism diagnostic criteria and patient exclusion criteria 2

Population Genetics Analysis 2

Statistical analysis 7

Supplementary Figures 8

Supp. Figure S1. Simulating non-uniform recombination and mutation with *ms*. 8

Supp. Figure S2. Simulation of haplotype decay by recombination. 9

Supp. Figure S3. Density plots of distributions of tMRCA and of the number of R304* allele carriers per generation predicted by forward simulation. 11

Supplementary Tables 12

Supp. Table S1. Haplotype data of Irish R304* pedigrees 12

Supp. Table S2. Intermediate results. 13

Supp. Table S3. Genotyping results and clinical details of Mid Ulster screening subjects 14

Supp. Table S4. Clinical details of Irish R304* pedigrees. 15

Supp. Table S5. Distribution of AIP variants identified in 116 Irish patients with somatotrophinomas 16

Supp. Table S6. Clinical details of Irish AIP-negative FIPA pedigrees 18

Supp. Table S7. Details of historical Irish giants, including AIP genotyping results. 19

Supplementary References 20

Supplementary Methods

Sample size calculation

The calculated sample size for the Mid Ulster screening study was 804, for a power of 0.8 and alpha of 0.05, assuming the estimated carrier frequency (2/1000, based on preliminary data) would be significantly higher than a null frequency of 1/10000 or less (a conservative estimate of zero frequency); Greater Belfast and ROI general population samples were size-matched with the Mid Ulster sample.

Mid Ulster screening and genotyping of population control groups

The Mid Ulster screening volunteers (n=936) were recruited at collection points in the local towns of Cookstown and Dungannon, following extensive advertisement in the local media, and provided information about their addresses, personal and family history of pituitary adenoma and tall stature (defined for screening participants as height ≥ 180 cm for females and ≥ 198 cm for males). Two large population control groups were genotyped for R304*, one from the Greater Belfast region in Northern Ireland (NI), previously recruited in the PRIME study (n=1000) (Linden, et al., 2012), the other from the ROI (n=2094) (Table 1); no additional information about the place of birth / residence of these controls was available. R304* genotyping was performed by Kaspar (LGC Genomic Solutions, Hoddesdon, UK) or TaqMan® allele-specific assays (#4332072 TaqMan® SNP Genotyping Assay, Life Technologies, Paisley, UK). Genotyping included positive (heterozygous R304*) and negative (wild-type and no template) controls; R304*-carriers were confirmed by dye-terminator sequencing. Screening-detected carriers and their relatives underwent genetic counselling and confirmatory or predictive genetic testing. Carriers were invited for endocrine evaluation.

Gigantism diagnostic criteria and patient exclusion criteria

Gigantism was diagnosed in patients with somatotrophinomas meeting at least one of the following criteria: somatotrophinoma onset before 18 years of age, height ≥ 3 standard deviations (SD) above mean for sex and age, or ≥2 SD over calculated midparental height (Hernández-Ramírez, et al., 2015). Multiple endocrine neoplasia type 1 was excluded clinically in all patients and *MEN1* mutations were absent in selected patients who were tested. One Carney complex patient (*PRKAR1A* mutation-positive) was excluded.

Population Genetics Analysis

We estimated two quantities through population genetics analysis: the tMRCA of the haplotypes that encompass the R304* allele, and conditioned on the tMRCA, the total number of carriers expected at present time. Fourteen microsatellite markers (short tandem repeats – STR) covering 8.3

Mbp in the genomic vicinity of *AIP* were genotyped in at least one R304* carrier in each pedigree, as previously described (Chahal, et al., 2011). Haplotypes were computed using PHASE (Stephens, et al., 2001), incorporating prior phasing information deduced from closely-related allele carriers (three pedigrees), and the conserved ancestral haplotype around *AIP* was established manually (Supp. Table S1). All R304* carriers were heterozygous; homozygous *AIP* disease alleles lead to embryonic lethality in animal models (Lin, et al., 2007; Raitila, et al., 2010). The haplotypes are shaped by two processes, STR mutations and recombination. Our samples show no evidence of STR mutation, but indicate a number of recombination events leading to a decay of the ancestral haplotype (light grey shading, Supp. Table S1) around the c.910C>T mutation. The ancestral haplotype remains fully conserved among the 18 Irish individuals only between the two markers directly flanking the AIP gene (dark grey shading, Supp. Table S1).

At least two classes of haplotype-based approaches have previously been described to bound the age of a mutational event by estimating the tMRCA from a sample of chromosomes. The first class relies on the fact that linkage disequilibrium with nearby alleles decays exponentially with generations at a rate proportional to the recombination rate, a principle that has been coined "genetic clock". Several variants of the basic approach have been proposed (see Colombo, 2007 and references therein) (Colombo, 2007), many of which require genotyping markers around the locus of interest in both carriers and non-carriers of the relevant allele (see for example Stephens, et al., 1998) (Stephens, et al., 1998). As we only have genotype data from individuals carrying the R304* allele, the latter group of approaches is not applicable here. The second class estimates the tMRCA of the haplotype carrying the allele of interest in an explicit coalescence theory framework, which is the approach followed in Chahal *et al.*, 2011. The shortcoming of this analytical solution however is that it requires the considered region to be fully conserved in all chromosomes. Therefore, it is not able to take into account the additional information in haplotype regions conserved in only part of the samples. A method fully integrating the information contained in the observed haplotype patterns has been proposed (Austerlitz, et al., 2003). However, the approach assumes independence

between samples, i.e. a star-like genealogy, which is corrected for in the case of the tMRCA estimate itself but does not allow to derive meaningful confidence intervals as these strongly depend on genealogy.

In order to address these problems, we devised a new strategy to calculate the tMRCA of the given haplotypes with the R304* allele. We followed a mixed approach combining coalescence theory and simulations in an Approximate Bayesian Computation framework (Beaumont, et al., 2002). Analogous to the method we previously used (Chahal, et al., 2011), we first exploited the presence of a region fully conserved in all individuals which allowed to analytically compute the expected tMRCA of a set of samples, given only that they share this region. This calculation, based on coalescence theory is presented in Donnelly *et al.* 1996 (Donnelly, et al., 1996)

(1)

$$\mathbb{E}[T|D] = N \sum_{i=2}^{|D|} \frac{2}{i(i-1+2Nr)}$$

where T is the tMRCA in number of generations, D is the observed data consisting of $|D|$ samples that lack variation with respect to a mutational process that occurs at rate r per generation, and N is the effective population size. In comparison,

(2)

$$\mathbb{E}[T] = 4N\left(1 - \frac{1}{|D|}\right)$$

is the expectation for $|D|$ samples from the standard coalescent model not conditioned on D (see e.g. page 138 in Templeton, 2006(Templeton, 2006)). In Chahal *et al.,* 2011 (Chahal, et al., 2011), a pooled STR mutation and recombination rate $r = mr_m + r_c$ was used, where m is the number of conserved STRs, r_m is the STR mutation rate per generation assumed to be 0.001, and r_c is the recombination rate per generation obtained by Haldane's map function

(3)

$$r_c = \frac{1 - \exp(-2d)}{2}$$

where d is the genetic distance between the markers delimiting the conserved region in Morgans. Subsequently, assume an effective population size of $N = 10^4$ and $|D| = 17$ (the number of contemporary pedigrees sharing a common ancestor). We applied Equation (1) on the region between markers D11S1249 and D11S1889. The corresponding mutation rate is a sum based on $m = 2$ and the genetic distance between the two markers which we obtain either from the HapMap (The HapMap International Consortium, 2007) or the Rutgers Combined Linkage Physical Map (Matise, et al., 2007) (Supp. Table S1). A full list of intermediate results is provided (Supp. Table S2). These values represent a first estimate for the tMRCA. However, this approach does not make full use of the data, as it ignores partial haplotype conservation around the strictly conserved regions and leads to a large overestimation of the tMRCA (Supp. Table S2, row E). Therefore, we complemented the analytical calculations by a simulation-based approach, incorporating the above results as priors. We phrased the problem within the ABC framework: we simulated genealogies based on the coalescent process with recombination and mutation, and obtained a distribution of tMRCA from those simulations that best reproduced the pattern of haplotype conservation and STR mutation observed in the data.

A set of observed or simulated haplotypes can be represented by a vector of counts (c_1,…, c_n), where c_i is the number of individuals that share the ancestral haplotype around the central mutation up to the ith marker. Note that this is not a lossless representation of the data, but a summary statistic as is common in ABC. For example, in this notation our data from (Supp. Table S1) becomes (6, 8, 9, 14, 15, 16, 17, 17, 17, 16, 15, 15, 15, 14, 10). This allows to define a distance d between a simulation $S = (s_1, \ldots, s_n)$ and the observed data O = (o_1, …, o_n) as

(4)

$$d(S,O) := \sum_i (o_i - s_i)^2$$

where *i* iterates over the relevant markers. We performed 10^7 simulations using the program *ms* (Hudson, 2002). We fixed an effective population size of $N = 10^4$, and modelled both recombination and mutation with the mutation parameter Θ of *ms* (Supp. Figure S1). This is not generally correct and only possible here because we assumed that all recombinations were with unobserved haplotypes and the relevant information for further analysis was determined only by the nearest recombinations to either side of *AIP*. The mutation rates were scaled in order to incorporate the prior information on the tMRCA derived from Equation (1). Scaling factors are given as supporting information (Supp. Table S2, row F). We only accepted a simulation if it exactly matched the mutational pattern in the data, that is no STR mutation. The remaining simulations were ranked according to the distance defined in Equation (4). Simulation results are exemplified as figures (Supp. Figures 2*A* and 2*B*). Finally, we applied regression adjustments to simulation results, to obtain adjusted tMRCA distributions (Supp. Figure S2*C*). The approach described here is a powerful way to estimate the tMRCA of the given haplotypes, compared to methods used previously (Chahal, et al., 2011); however, there are several limitations to our analysis. Most importantly, we did not model the effect of ascertainment on the *AIP* mutations. Furthermore, owing to our ABC approach we did not consider the full data but only summary statistics.

Given distributions of tMRCA of R304* allele haplotypes (Supp. Figures 3*A* and 3*B*), we estimated the expected number of carriers today using the forward simulation approach presented in Chahal *et al.*, 2011 (Chahal, et al., 2011). Let f_g and N_g denote the relative allele frequency *f* and population size *N* at generation *g*. Every simulation starts with a single carrier, i.e. with an allele frequency $f_1 = \frac{1}{N_1}$. Population growth was incorporated by iterative updating, $N_{g+1} = (1 + \frac{p}{100})N_g$, leading to an exponential population growth of *p* per cent each generation. Lastly, the allele frequency at

generation *g+1* was obtained by binomial sampling taking population growth into account, $f_{g+1} = B(N_{g+1}, f_g)$.

We performed 10^6 simulations starting with an initial population size $N_1 = 10^4$ growing 2% each generation, *i.e.* $p = 2$. We took the uncertainty about the tMRCA into account by sampling the duration of a simulation from the tMRCA distributions established previously. Finally, we conditioned the resulting distribution of number of carriers per generation (Chahal, et al., 2011) in the present population to values greater than or equal to 27, accounting for the prior knowledge from direct observation (Supp. Figures 3*C* and 3*D*).

Statistical analysis

Statistical analysis of clinical and biochemical data used JMP v.9 software (SAS Institute, Cary, USA). Continuous variables were tested for normality with the Shapiro-Wilk test, reported as median (range or 95% confidence interval [CI]) or mean (± SD) and tested using Mann-Whitney U or Student's t tests. Categorical variables, reported as counts (percentages) were tested using Fisher's exact test. Allele frequency estimate confidence intervals were computed using the Beta distribution with a Jeffreys prior for non-zero observed (implemented in the qbeta R function) and a previously described method for zero observed (Burger, et al., 2007). The probability that X>Y for two distributions of tMRCA estimates has been obtained as the proportion of times X>Y in 10^6 pairs of samples from the distributions.

Supplementary Figures

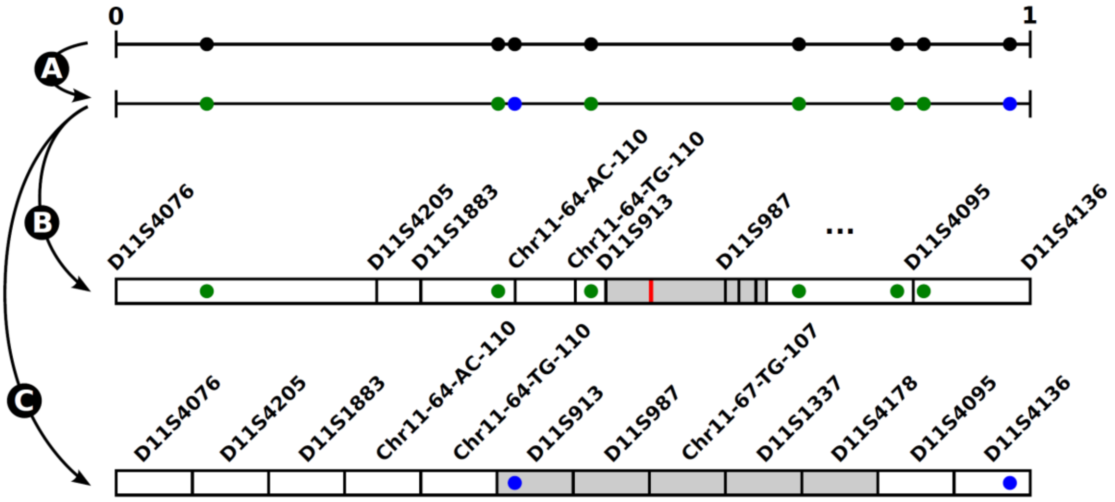

Supp. Figure S1. Simulating non-uniform recombination and mutation with *ms*.
We fixed a pooled mutation rate *r* using the option '-t' of *ms* (Hudson, 2002), resulting in genealogies with initially ambiguous mutational events mapped onto the interval [0, 1]. In a first step (A), we colour the mutational events to be either recombinations (green) or STR mutations (blue) by a series of Bernoulli draws with probability defined by the proportion of recombination to mutation rate (Supp. Table S2, row J). Second, the positions of recombination and mutation events are mapped from the interval [0; 1] onto the chromosome. For recombination (B), the relative genetic distance between the markers is taken into account (Supp. Table S1). The mapping for STR mutations (C) is simpler as a uniform STR mutation rate of 0.001 is assumed leading to an equally spaced partition, however, differences in mutation rate could easily be incorporated. Note that the region between markers D11S1249 and D11S1889 (red line) is excluded for both recombination and mutation, as it has already been taken into account in the prior analytical calculations. The area shaded in grey represents the region that is inferred to have conserved the ancestral haplotype. Finally, note that this specific simulation would be rejected as it carries a mutation in D11S913 not present in the observed data (we excluded the rare possibility of a microsatellite mutation reverting a recombinant allele back to its original size). The mutation in D11S4136 is not within the conserved haplotype (grey area) and is therefore ignored in the analysis.

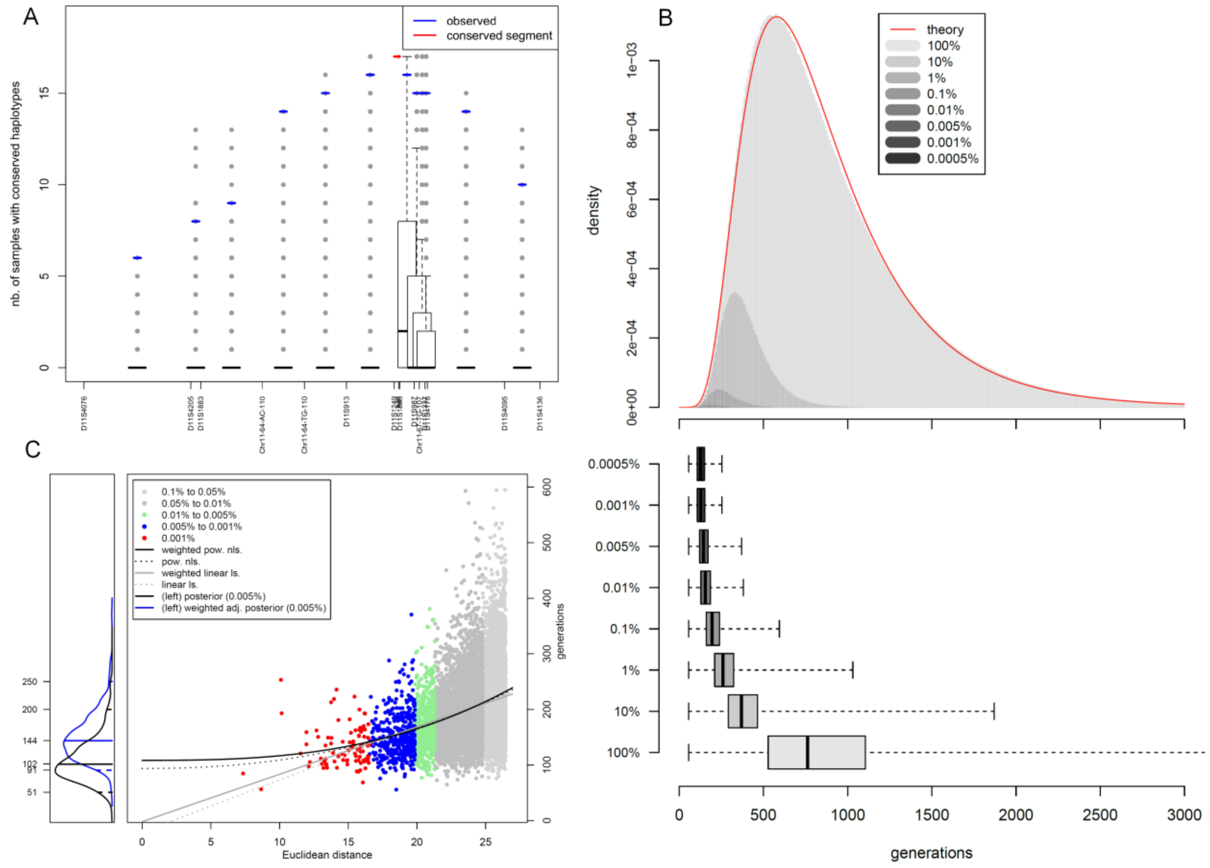

Supp. Figure S2. Simulation of haplotype decay by recombination.

(A) Summary of 10^7 simulations performed with *ms* (Hudson, 2002) and the observed pattern of haplotype conservation (in blue). The actual number of accepted simulations was 9950227 (lower than 10^7) as the observed absence of STR mutation had to be matched. Recombination rates were calculated according to Rutgers maps genetic distances. Each simulation corresponds to one value per inter-marker region; simulations are represented as vector of counts (Supp. Methods). The red segment is excluded from the simulations as it is fully conserved in all individuals and used to derive the analytical prior (Supp. Methods). Most of the boxplots show as lines because recombination events occur in the majority of the simulations and none of the samples conserve the ancestral haplotype. Outliers (grey points) are plotted on top of each other and may therefore represent multiple points. (B) tMRCA distribution of simulations. Upper panel shows the theoretical expectation which is a log-normal curve with mean calculated by Equation (2) and variance $\sum_{i=2}^{|D|} \frac{16N^2}{i^2(i-1)^2}$, see e.g. page 138 in Templeton (Templeton, 2006) with all variables defined as in Equation (2). Lower panel shows the distribution of simulated results. The distributions in darker grey are obtained by successively restricting the set of simulations to those that best match the observed haplotype pattern, i.e. to those with smallest distance as defined in Equation (4). (C) Final distribution of tMRCA (only HapMap-based results shown). We applied regression adjustments, as

previously described (Donnelly, et al., 1996), fitting regression curves by linear/non-linear least squares on the 0.005% closest simulations in terms of the distance defined in Equation (4). Four regressions are shown: both weighted (solid) and unweighted (dashed) linear (grey; $y = a + bx$) and power (black; $y = a + bx^c$) models. A likelihood ratio test confirmed that the weighted power model provided a better fit ($p < 10^{-10}$) than the linear one. Vertical left panel shows the distributions of unadjusted (blue), and of weighted power model-adjusted age estimates (black), reporting the 2.5th, 50th and 97.5th quantile. Abbreviations: power model (pow.), non-linear least squares (nls.), adjusted (adj.)

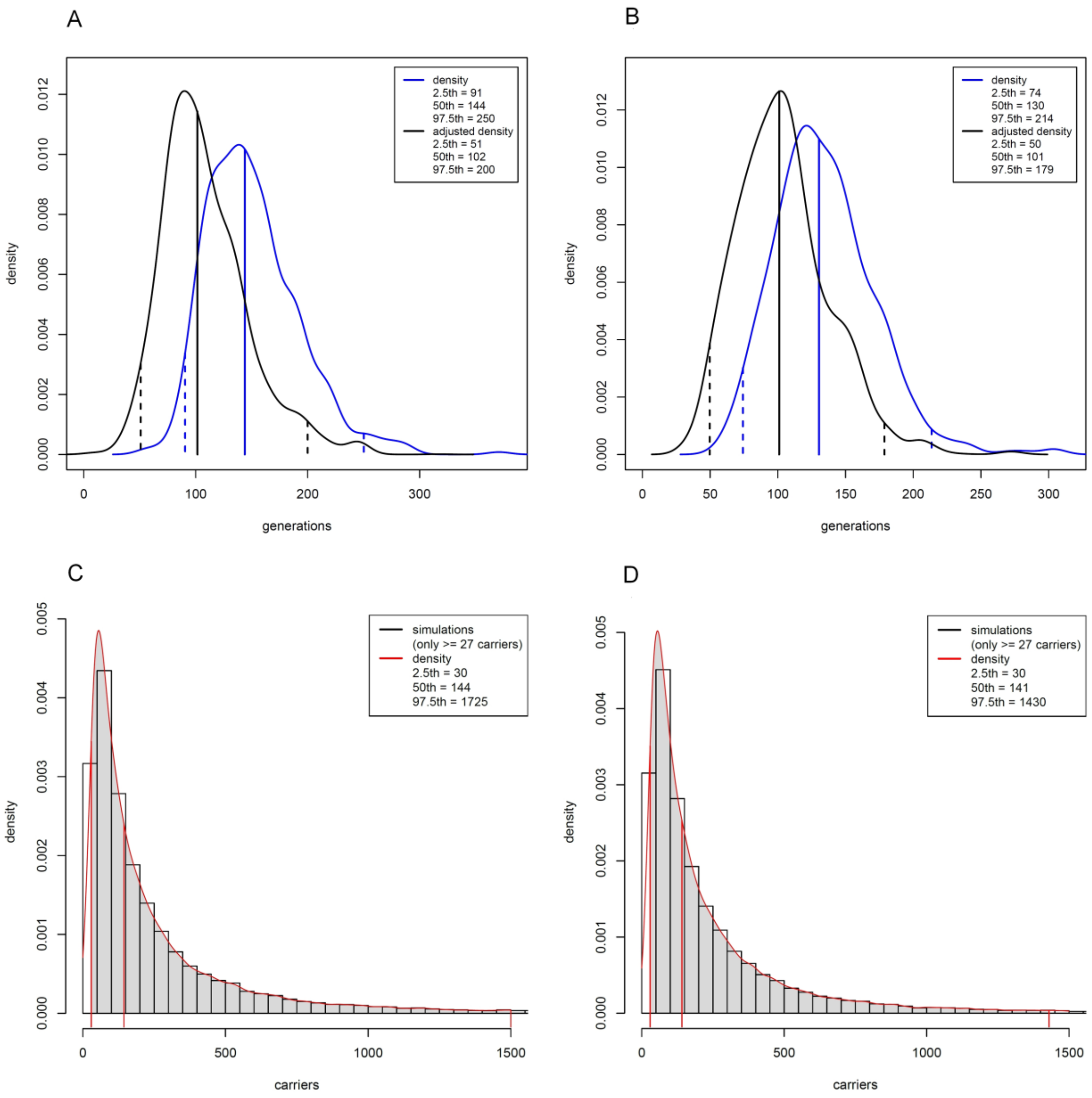

Supp. Figure S3. Density plots of distributions of tMRCA and of the number of R304* allele carriers per generation predicted by forward simulation.

tMRCA (A, B) shown as tree height, in generations, for the *ms* coalescent-simulated trees best-fitting the haplotype conservation seen in the Irish founder pedigrees. Weighted power-adjusted (black) and non-adjusted (blue) tMRCA distributions are shown; Simulation distribution of numbers of carriers (C, D). Estimates were calculated according to HapMap (A, C) or Rutgers (B, D) genetic map distances.

Supplementary Tables

Supp. Table S1. Haplotype data of Irish R304* pedigrees

	D11S4076	D11S4205	D11S1883	Chr11-64-AC-110	Chr11-64-TG-110	D11S913	D11S1249	*AIP* genotype	D11S1889	D11S987	Chr11-67-TG-107	D11S1337	D11S4178	D11S4095	D11S4136
start position (bp)	61363095	63182852	63373724	64500839	65211712	65936161	67106653	67250505	67313128	67893341	68004998	68131658	68189101	69268143	69615686
end position (bp)	61363349	63183185	63374007	64500948	65211821	65936492	67106843	67258579	67313325	67893449	68005104	68132045	68189409	69268378	69615920
HapMap distance (cM)	68.87	70.83	71.16	71.87	72.32	72.55	72.86	72.89	72.91	73.45	73.55	73.68	73.76	74.86	75.74
Rutgers distance (cM)	72.02	73.72	73.88	74.86	75.53	76.2	76.96	77.03	77.05	77.28	77.36	77.45	77.49	78.72	79.28
**18th century patient**	-	-	15	17	26	10	-	R304*	34	13	21	-	-	-	-
	-	-	14	20	25	10	-	wt	38	16	22	-	-	-	-
**FIPA 1**	16	19	20	22	26	10	19	R304*	34	13	22	48	80	22	15
	19	17	14	22	17	10	20	wt	37	20	19	48	80	22	19
**FIPA 2**	16	19	20	22	26	10	19	R304*	34	13	21	52	80	25	20
	16	17	16	19	28	9	17	wt	29	16	22	48	80	22	18
**FIPA 3**	20	17	16	22	30	10	19	R304*	34	13	21	52	80	25	20
	18	17	18	21	31	9	21	wt	38	14	23	47	75	22	24
**FIPA 4**	21	19	20	22	26	10	19	R304*	34	13	21	52	80	25	19
	21	18	22	17	28	9	18	wt	35	14	23	48	81	25	19
**FIPA 5**	19	17	14	22	26	10	19	R304*	34	16	21	48	80	25	20
	20	17	13	21	25	11	18	wt	37	17	22	49	80	22	20
**FIPA 6**	20	17	16	22	26	10	19	R304*	34	13	21	52	80	25	20
	22	17	17	21	26	9	18	wt	38	18	25	49	83	22	15
**FIPA 7**	16	19	20	22	26	10	19	R304*	34	13	21	52	80	25	20
	16	17	16	21	23	11	18	wt	37	14	22	48	80	25	20
**FIPA 8**	20	17	15	20	26	10	19	R304*	34	13	21	52	80	20	16
	20	15	19	21	17	9	16	wt	38	17	22	48	80	27	20
**SP 1**	16	17	16	22	26	10	19	R304*	34	13	21	52	80	25	20
	19	17	15	20	23	9	18	wt	37	14	23	48	80	22	16
**Sp 2**	16	17	20	22	26	10	19	R304*	34	13	21	52	80	25	20
	16	17	17	25	26	9	18	wt	29	12	22	49	82	25	15
**Sp 3**	19	17	14	21	24	9	19	R304*	34	13	21	52	80	25	20
	19	16	14	23	27	8	22	wt	37	14	20	55	72	25	24
**Sp 4**	18	19	20	22	26	10	19	R304*	34	13	21	52	80	25	20
	21	15	21	21	29	11	18	wt	29	15	22	48	82	25	23
**Sp 5**	20	17	15	22	26	10	19	R304*	34	13	21	52	80	25	15
	20	17	16	17	24	11	18	wt	37	17	18	51	79	22	19
**Sp 6**	16	19	20	22	26	10	19	R304*	34	13	21	52	80	25	20
	20	19	14	22	26	10	20	wt	37	21	22	47	80	22	20
**Sp 7**	16	17	16	22	26	10	19	R304*	34	13	21	52	80	25	16
	14	18	17	21	22	10	18	wt	37	17	23	52	83	22	22
**Screening 1**	16	19	20	22	26	10	19	R304*	34	13	21	52	80	25	19
	20	17	14	21	17	10	19	wt	39	17	22	48	80	25	21
**Screening 2**	16	19	20	22	26	10	19	R304*	34	13	21	52	80	25	20
	20	17	17	21	29	9	16	wt	38	17	21	48	80	25	21

Both physical positions (in base pairs, bp) and genetic distances (in centimorgans, cM) refer to human genome version hg19. All genetic distances are sex-averaged and were obtained from the HapMap project combining all populations and from the Rutgers Combined Linkage-Physical Map. Rutgers distances are smoothed values, generated by fitting local quadratic curves in order to eliminate distances of 0 cM that result from lack of power to detect recombination events in small map interval. Alleles are reported as number of repeats. Dark grey shading = core haploblock conserved in all pedigrees; light grey = additional haplotype blocks shared between pedigrees. wt = wildtype allele; '-' = missing data, due to genotyping failure in the ancient DNA samples. R304* = NM_003977.3:c.910C>T

Supp. Table S2. Intermediate results.

	Description	HapMap	Rutgers
**A**	full region (cM)	6.87	7.26
**B**	conserved region (cM) (Supp. Table S1)	0.05	0.09
**C**	μ of conserved region (Supp. Methods)	0.0025	0.0029
**D**	expectation tMRCA (generations) (see Equation [2])	37647	37647
**E**	conditioned expectation tMRCA (generations; see Equation [1])	883	771
**F**	scaling factor $\left(\frac{D}{E}\right)$	42.61	48.81
**G**	variable region (cM) (Supp. Table S1)	6.82	7.17
**H**	μ of variable region	0.7575	0.788
**I**	scaled μ of variable region $\left(\frac{H}{F}\right)$	0.0018	0.0016
**J**	Proportion $\frac{r_c}{\mu}$	0.08416	0.08477

tMRCA = time to the most recent common ancestor

Numerical values of quantities computed from the haplotype data (Supp. Table S1). In all cases an effective population size of 10^4 is assumed. Deviations in ratios above are due to rounding.

Supp. Table S3. Genotyping results and clinical details of Mid Ulster screening subjects

AIP genotype (no. of subjects)	Family history of PA (no. of subjects)	Subject details (gender, age - years, diagnosis, family history, endocrine test results)
**R304* / wt (6)**	Positive FH (3)	- M, 65, possible microadenoma on pituitary MRI, slight IGF-I increase (1·17 x ULN), normal GH-OGTT, FH of FIPA (FIPA 1)<br>- F, 46, gigantism at 17 years, FH of R304*-positive FIPA (FIPA 4), previously not tested for *AIP* mutations (had been lost to follow-up)<br>- M, 37, clinically unaffected, relative of R304*-positive patient (Sp4 pedigree), did not attend endocrine assessment
	Negative FH (3)	- M, 42, known R304*-positive acromegaly at 19 years (Sp4 pedigree)<br>- F, 35, clinically unaffected (Screening 1 pedigree) normal random GH and prolactin[a]<br>- M, 76, related to subject above (Screening 1), normal pituitary MRI, serum GH and prolactin[a]
**wt / wt (930)**	Positive FH (70)	- six members of R304*-positive FIPA pedigrees (FIPA1, 2 and 4). Results confirmed by diagnostic testing<br>- 45 relatives of 30 sporadic PA patients (10 acromegaly, four Cushing's disease, one NFPA, one PRL, 14 unspecified)<br>- 19 FH of very tall stature (adults ≥ 2SD height - 198 cm for males, 180 cm for females; children ≥3 SD height SD for age/gender)
	Negative FH (860)	- seven PA patients: three *AIP*mut-negative acromegaly (previous diagnostic testing - Belfast cohort), two NFPA, two Cushing's disease<br>- three with clinical suspicion of acromegaly[b]: two had subsequent normal biochemical screening (IGF-I, prolactin and random GH), no MRI, one did not attend endocrine assessment<br>- three very tall individuals without clinical signs of acromegaly, not assessed further: F, 34, 186 cm, M, 46, 198 cm, M, 41, 198 cm

R304* = NM_003977.3:c.910C>T, F = female, M = male, FH = family history, PA = pituitary adenoma, ULN = upper limit of normal,

[a] These two subjects represent a novel R304* pedigree: Screening 1 (Figure 2 and Supp. Table S4); three additional clinically unaffected carriers were identified through cascade genetic testing: F, 26, no obvious signs of acromegaly, endocrine assessment not yet performed, M, 67, normal IGF-I, PRL, random GH, normal MRI and F, 72, normal IGF-I, PRL, GH-OGTT, normal pituitary MRI

[b] An additional subject suspected of gigantism (M, 25 years, acromegalic appearance, 204 cm tall, shoe size UK 14, R304*-negative), excluded from screening study due to non-Irish origin, was advised to seek medical attention (unknown outcome - lost contact)

Supp. Table S4. Clinical details of Irish R304* pedigrees.

No.	Pedigree ID	Pituitary adenoma clinical phenotype	No. of affected individuals	Affected individuals details (gender, age at onset – years, tumour size, diagnosis)	No. of unaffected carriers	Unaffected carriers details (gender, age at evaluation - years)
1	18th century patient (Chahal, et al., 2011; Stals, et al., 2011)	nk	1	M, nk, Macro, Gigantism	-	-
2	FIPA 1 (A) (Chahal, et al., 2011; Stals, et al., 2011)	GH/NFPA	8	F, 13, Macro, Gigantism<br>M, 16, Macro, Gigantism<br>F, 29, Micro, Acromegaly<br>F, 30, Micro, NFPA<br>F, 32, Macro, Gigantism<br>M, 33, Micro, NFPA<br>M, 36, no visible tumour, mildly elevated IGF-1<br>M, 68, Macro, Acromegaly	12	M, 3; F, 9; F, 25; F, 30; F, 35; F, 37; M, 39; M, 58; F, 59; M, 65; M, 67; M, 71;
3	FIPA 2 (B) (Chahal, et al., 2011; Stals, et al., 2011; Williams, et al., 2014)	GH/mixed GH-PRL	4	M, 13, Macro, Gigantism<br>F, 16, Macro, Mixed GH-PRL adenoma<br>M, 18, Macro, Acromegaly<br>M, 23, Macro, Acromegaly	16	F, 4; F, 5; F, 6; F, 7; M, 7; M, 8; M, 30; M, 32; F, 35; F, 38; M, 42; M, 42; M, 44; M, 69; M, 72; M, 73
4	FIPA 3 (E) (Stals, et al., 2011)	PRL/mixed GH-PRL	2	F, 21, Macro, Prolactinoma<br>F, 33, Macro, Mixed GH-PRL adenoma	-	-
5	FIPA 4	Isolated GH	2	M, 14, Macro, Gigantism<br>F, 17, Macro, Gigantism	1	F, 93
6	FIPA 5	Isolated GH	4	M, 15, nk, Gigantism<br>M, 15, nk, Gigantism<br>F, 17, Macro, Acromegaly<br>M, nk, nk, Gigantism	-	-
7	FIPA 6 (C) (Chahal, et al., 2011; Stals, et al., 2011)	GH/PRL/ mixed GH-PRL	5	F, 6, Macro, Gigantism<br>M, 15, Macro, Gigantism<br>F, 16, Macro, Prolactinoma<br>F, 32, Macro, Mixed GH-PRL adenoma<br>M, 62, Macro, Prolactinoma	5	M, 10; F, 14; M, 18; M, 47; M, 76

No.	Pedigree ID	Pituitary adenoma clinical phenotype	No. of affected individuals	Affected individuals details (gender, age at onset – years, tumour size, diagnosis)	No. of unaffected carriers	Unaffected carriers details (gender, age at evaluation - years)
8	FIPA 7 (D) (Stals, et al., 2011)	GH	2	M, 15, Macro, Gigantism<br>M, 17, Macro, Acromegaly	5	F, 39; M, 42; M, 53; F, 70; M, 88
9	FIPA 8	GH/PRL	2	M, 17, nk, Gigantism<br>M, 31, Macro, Prolactinoma	-	-
10	Sp 1	GH	1	M, 16, Macro, Gigantism	-	-
11	Sp 2	GH	1	M, 14, Macro, Gigantism	1	M, 63
12	Sp 3	GH	1	M, 21, Macro, Acromegaly	1	M, 66
13	Sp 4	GH	1	M, 19, Macro, Acromegaly	1	M, 39
14	Sp 5	Mixed GH-PRL	1	M, 9, Macro, Gigantism	-	-
15	Sp 6	GH	1	F, 30, Macro, Acromegaly	-	-
16	Sp 7	Mixed GH-PRL	1	M, 21, Macro, Mixed GH-PRL adenoma	3	F, 17; M, 53; M, 54
17	Screening 1	na	0	na	5	F, 26; F, 35; M, 67; F, 72; M, 76
18	Screening 2	na	0	na	3	F, 42; M, 71; M, 77

R304* = NM_003977.3:c.910C>T, nk = not known, na = not applicable, GH = somatotrophinoma, PRL = prolactinoma, Mixed GH-PRL adenoma=GH and prolactin-secreting adenoma.

Capital letter identifiers between parentheses were used in previous reports (Chahal, et al., 2011; Stals, et al., 2011).

Obligate carriers of unclear clinical status (deceased/unavailable for clinical examination) were excluded.

Supp. Table S5. Distribution of AIP variants identified in 116 Irish patients with somatotrophinomas.

AIP genotype	Wild-type	NM_003977.3:c.3G>A (p.0?)	NM_003977.3:c.100-18C>T (p.?)	NM_003977.3:c.469-17T>C (p.?)	NM_003977.3:c.815G>A (p.Gly272Asp)	NM_003977.3:c.910C>T (p.Arg304*)
Reference SNP Cluster (dbSNP)	na	na	rs202156895	na	na	rs104894195
Pathogenicity	na	Yes[a]	VUS[b]	VUS[c]	VUS[d]	Yes
Belfast (n=87)	71	2	2	0	1	11
Dublin (n=29)	25	0	1	1	0	2

All carriers were heterozygous; na = not applicable; VUS = variant of uncertain clinical significance.

[a] This variant, ClinVar ID: 253315 and AIP LSDB MutationID: M389 (https://www.ncbi.nlm.nih.gov/clinvar/variation/253315/ and http://bit.ly/2caOphV) (Hernández-Ramírez, et al., 2015) affects the translation initiation codon, putatively leading to usage of a downstream out-of-frame start codon at c.89 position and termination at c.464 stop codon; predicted protein sequence: MGPRPRSTTGRCTVTTRAPCWTTAGLVASPWSSSLARSSSCLCGRPSCAPCEKGRLPSSSVTSS MWSCTRWWPRVSATSRWARTPWRAS GTAAVLHRCVNTAPWAMLTWTPCSRTPSPSSSTWRC

[b] The pathogenicity of this variant has not been definitively established. It has been described in young-onset acromegaly patients (Georgitsi, et al., 2007; Hernández-Ramírez, et al., 2015; Leontiou, et al., 2008; Oriola, et al., 2013; Tichomirowa, et al., 2011) and up to 1.4% of European-ancestry individuals in variant databases

[c] Novel VUS, ClinVar ID: 253316 and AIP LSDB MutationID: M390 (https://www.ncbi.nlm.nih.gov/clinvar/variation/253316/ and http://bit.ly/2cgViKw)

[d] Novel VUS, also identified as rs779831121 by the ExAC project

Supp. Table S6. Clinical details of Irish AIP-negative FIPA pedigrees.

No.	FIPA phenotype	No. of affected individuals	Gender, age at onset (years.), tumour size, diagnosis
1	Isolated GH	2	M, 26, nk, Acromegaly<br>M, 39, Macro, Acromegaly
2	Isolated GH	2	F, 21, Macro, Acromegaly<br>M, 28, nk, Acromegaly
3	Isolated GH	2	M, 36, Macro, Acromegaly<br>M, 51, Macro, Acromegaly
4	PRL	2	M, 16, Macro, Prolactinoma<br>M, 20, Macro, Prolactinoma[a]
5	ACTH	2	F, 22, nk, Cushing's<br>F, 35, nk, Cushing's
6	NFPA	3	F, 35, micro, NFPA<br>M, 37, Macro, NFPA<br>M, nk, nk,nk
7	NFPA	2	M, 57, nk, NFPA<br>F, 83, nk, NFPA
8	NFPA/GH	2	M, 82, Macro, NFPA<br>M, nk, nk, Acromegaly
9	NFPA/GH	2	M, 60, Macro, NFPA<br>M, nk, nk, Acromegaly
10	PRL/GH	2	F, 23, Macro, Prolactinoma<br>M, 64, nk, Acromegaly
11	PRL/GH	2	F, 28, nk, Prolactinoma<br>nk, nk, nk, Acromegaly
12	ACTH/GH	2	F, 12, Macro, Cushing's disease<br>F, 48, nk, Acromegaly
13	PRL/NFPA/ GH	3	F, 47, nk, Prolactinoma<br>M, 51, Macro, NFPA<br>M, nk, nk, Gigantism

nk = not known, GH=somatotrophinoma, PRL=prolactinoma, Mixed GH-PRL adenoma=GH and prolactin-secreting adenoma, NFPA=non-functioning pituitary adenoma, ACTH=Cushing's disease

[a] This patient presented the heterozygous *AIP* synonymous variant NM_003977.3:c.906G>A, (p.(=)), also described as rs142912418, while his affected brother did not harbour the variant. Lack of segregation and the synonymous nature suggest this variant is not pathogenic

Supp. Table S7. Details of historical Irish giants, including AIP genotyping results.

No	Name (dates of birth-death/date when observed)	Evidence	Height (cm)	*AIP*mut status	Place of birth
**1**	Mary Murphy (cca. 1696)	'The Portrush Giantess', manuscript of James Paris du Plessis (British Library, Sloane Manuscript 3253)	213	unknown	Portrush, Co. Antrim
**2**	James Kirkland (cca. 1730)	Member of Potsdam Giants guard "Lange Kerls" (portrait by Johann Christof Merk)	217	unknown	Ballygar<br>Co. Galway
**3**	Cornelius Magrath (1736-1760)	Skeleton at Trinity College, Dublin (Cunningham, 1892; Cunningham, 1902)	226.1	wild-type	Tipperary, Co. Tipperary
**4**	Thomas Fanton (1745-1782?)	The Hibernian Giant, according to contemporary newspapers	unknown	unknown	Omagh<br>Co. Tyrone
**5**	Charles Byrne (1761-1783)	Skeleton in Hunterian museum, London (Bergland, 1965; Chahal, et al., 2011)	231	R304*-positive	Littlebridge, Drummullan<br>Co. Tyrone[b]
**6-7**	Knipe brothers (cca. 1760-?)	Identical twins, cousins of Charles Byrne (Bergland, 1965; Chahal, et al., 2011)<br>Etching by John Kay, National Portrait Gallery, London	218.4	Assumed R304*-positive	Magherafelt<br>Co. Londonderry[b]
**8**	Patrick Cotter (1760-1806)	Examination (Fawcett, 1909; Frankcom and Musgrave, 1976), skeleton cremated in 1986[a]<br>Age of diagnosis of abnormal stature 17y	246.3	unknown	Kinsale<br>Co. Cork
**9**	Big Magee (cca. 1800)	Book description (Carleton, 1996; Lynass, 1842)	unknown	unknown	Clogher, Co. Tyrone[b]
**10**	Hugh Murphy (1842-1875)	Historical, photographs	217.2	unknown	Waterford, Co. Waterford
**11**	Simon McCann (1855-1900)	Contemporary Living magazine, May 2008	223.5	Assumed R304* (FIPA 5 pedigree)	Mountain Lodge<br>Co. Cavan
**12**	Patrick Murphy (1834-1862)	Historical, photographs	222	Unknown	Killowen<br>Co. Down
**13**	nk (cca. 1870)	Grandfather of Jim Cully (no. 19 below), St. Petersburg Times, USA, 19th April 1948 newspaper article and interview	223.5	Assumed R304* (FIPA 7 pedigree)	Co. Tipperary
**14**	Bernard McCrystal (1884-1905)	Relative of Mid Ulster screening participant	213.4	unknown	Omagh, Co. Tyrone
**15**	John Johnston (1898-1918)	Relative of Mid Ulster screening participant, Daily Sketch (Canadian Newspaper), 1918 May 13th May (Figure 3*A*)	211	unknown	Maghera<br>Co. Londonderry[b]
**16**	James McCooke (cca. 1890-?)	Relative of Mid Ulster screening participant Garvagh museum photograph (Figure 3*B*)	213	unknown	Garvagh<br>Co. Londonderry[b]
**17**	Michael Grealish (1892 - ?)	The Toronto World newspaper, 1916 April 11th& May 5th	218.4	unknown	Bohola, Co. Mayo
**18**	nk (nk)	'The Cruit Island giant', skeleton of a late adolescent, uncovered in 1954 on Cruit Island, Donegal Democrat newspaper, 2014 Feb 6th	236	unknown	Sean Baile, Co. Donegal
**19**	Jim Cully (1921-1970)	Family history, photographs	218.4	Assumed R304* (FIPA 7 pedigree)	Co. Tipperary, family from Northern Ireland
**20**	J.C. (1932-1969)	Prezio et al. (Prezio, et al., 1961) [c]	263.5	unknown	Unknown

R304* = NM_003977.3:c.910C>T

[a] Personal communication by Jonathan Musgrave (2011), who studied the skeleton in 1973

[b] Mid Ulster, including geographical cluster area

[c] Patient of Irish origin according to published case report. He showed rapid growth from very early childhood, this pattern may correspond to the X-LAG syndrome (Trivellin, et al., 2014) rather than to *AIP*mut-related gigantism

Supplementary References

Austerlitz F, Kalaydjieva L, Heyer E. 2003. Detecting population growth, selection and inherited fertility from haplotypic data in humans. Genetics 165(3):1579-86.

Beaumont MA, Zhang W, Balding DJ. 2002. Approximate Bayesian computation in population genetics. Genetics 162(4):2025-2035.

Bergland RM. 1965. New information concerning the Irish giant. J Neurosurg 23(3):265-9.

Burger J, Kirchner M, Bramanti B, Haak W, Thomas MG. 2007. Absence of the lactase-persistence-associated allele in early Neolithic Europeans. Proc Natl Acad Sci U S A 104:3736-3741.

Carleton W. 1996. William Carleton: the Autobiography: White Row Press Ltd.

Chahal HS, Stals K, Unterländer M, Balding DJ, Thomas MG, Kumar AV, Besser MG, Atkinson BA, Morrison PJ, Howlett TA, Levy MJ, Orme SM, et al. 2011. AIP mutation in pituitary adenomas in the 18th century and today. N Engl J Med 364(1):43-50.

Colombo R. 2007. Dating Mutations. eLS: John Wiley & Sons, Ltd.

Cunningham DJ. 1892. The skull and some of the other bones of the skeleton of Cornelius Magrath, the Irish giant. The Journal of the Anthropological Institute of Great Britain and Ireland 21:40-41.

Cunningham DJ. 1902. Cornelius Magrath, the Irish Giant. Man(112):156.

Donnelly P, Tavaré S, Balding DJ, Griffiths RC. 1996. Estimating the age of the common ancestor of men from the ZFY intron. Science 272(5266):1357-9; author reply 1361-2.

Fawcett E. 1909. Patrick Cotter-The Bristol Giant. The Journal of the Royal Anthropological Institute of Great Britain and Ireland 39(Jan-Jun 1909):196-208.

Frankcom G, Musgrave JH. 1976. The Irish Giant: Gerald Duckworth & Co Ltd.

Georgitsi M, Raitila A, Karhu A, Tuppurainen K, Mäkinen MJ, Vierimaa O, Paschke R, Saeger W, van der Luijt RB, Sane T, Robledo M, De Menis E, et al. 2007. Molecular diagnosis of pituitary adenoma predisposition caused by aryl hydrocarbon receptor-interacting protein gene mutations. Proc Natl Acad Sci U S A 104(10):4101-5.

Hernández-Ramírez LC, Gabrovska P, Dénes J, Stals K, Trivellin G, Tilley D, Ferrau F, Evanson J, Ellard S, Grossman AB. 2015. Landscape of Familial Isolated and Young-Onset Pituitary Adenomas: Prospective Diagnosis in AIP Mutation Carriers. J Clin Endocrinol Metab 100(9):E1242-54.

Hudson RR. 2002. Generating samples under a Wright-Fisher neutral model of genetic variation. Bioinformatics (Oxford, England) 18:337-338.

Leontiou CA, Gueorguiev M, van der Spuy J, Quinton R, Lolli F, Hassan S, Chahal HS, Igreja SC, Jordan S, Rowe J, Stolbrink M, Christian HC, et al. 2008. The role of the aryl hydrocarbon receptor-interacting protein gene in familial and sporadic pituitary adenomas. J Clin Endocrinol Metab 93(6):2390-401.

Lin BC, Sullivan R, Lee Y, Moran S, Glover E, Bradfield CA. 2007. Deletion of the aryl hydrocarbon receptor-associated protein 9 leads to cardiac malformation and embryonic lethality. The Journal of Biological Chemistry 282(49):35924-32.

Linden GJ, Linden K, Yarnell J, Evans A, Kee F, Patterson CC. 2012. All-cause mortality and periodontitis in 60-70-year-old men: a prospective cohort study. J Clin Periodontol 39(10):940-6.

Lynass B. 1842. Orange & Love Poems and Songs. Belfast.

Matise TC, Chen F, Chen W, De La Vega FM, Hansen M, He C, Hyland FCL, Kennedy GC, Kong X, Murray SS, Ziegle JS, Stewart WCL, et al. 2007. A second-generation combined linkage-physical map of the human genome. Genome Res 17:1783-1786.

Oriola J, Lucas T, Halperin I, Mora M, Perales MJ, Alvarez-Escolá C, Paz DM-N, Díaz Soto G, Salinas I, Julián MT, Olaizola I, Bernabeu I, et al. 2013. Germline mutations of AIP gene in somatotropinomas resistant to somatostatin analogues. Eur J Endocrinol 168(1):9-13.

Prezio JA, Griffin JE, O'Brien JJ. 1961. Acromegalic gigantism. The Buffalo giant. The American Journal of Medicine 31:966-76.

Raitila A, Lehtonen HJ, Arola J, Heliövaara E, Ahlsten M, Georgitsi M, Jalanko A, Paetau A, Aaltonen LA, Karhu A. 2010. Mice with inactivation of aryl hydrocarbon receptor-interacting protein

(Aip) display complete penetrance of pituitary adenomas with aberrant ARNT expression. The American Journal of Pathology 177(4):1969-76.

Stals K, Trivellin G, Korbonits M. 2011. AIP mutation in pituitary adenomas. N Engl J Med 364(20):1974-5.

Stephens JC, Reich DE, Goldstein DB, Shin HD, Smith MW, Carrington M, Winkler C, Huttley GA, Allikmets R, Schriml L, Gerrard B, Malasky M, et al. 1998. Dating the origin of the CCR5-Delta32 AIDS-resistance allele by the coalescence of haplotypes. Am J Hum Genet 62(6):1507-15.

Stephens M, Smith NJ, Donnelly P. 2001. A new statistical method for haplotype reconstruction from population data. Am J Hum Genet 68(4):978-89.

Templeton AR. 2006. Population Genetics And Microevolutionary Theory. Hoboken, New Jersey: John Wiley & Sons, Inc.

The International HapMap Consortium. 2007. A second generation human haplotype map of over 3.1 million SNPs. Nature 449:851-61.

Tichomirowa MA, Barlier A, Daly AF, Jaffrain-Rea ML, Ronchi C, Yaneva M, Urban JD, Petrossians P, Elenkova A, Tabarin A, Desailloud R, Maiter D, et al. 2011. High prevalence of AIP gene mutations following focused screening in young patients with sporadic pituitary macroadenomas. Eur J Endocrinol 165:509-515.

Trivellin G, Daly AF, Faucz FR, Yuan B, Rostomyan L, Larco DO, Schernthaner-Reiter MH, Szarek E, Leal LF, Caberg J-H, Castermans E, Villa C, et al. 2014. Gigantism and acromegaly due to Xq26 microduplications and GPR101 mutation. N Engl J Med:1-12.

Williams F, Hunter S, Bradley L, Chahal HS, Storr HL, Akker SA, Kumar AV, Orme SM, Evanson J, Abid N, Morrison PJ, Korbonits M, et al. 2014. Clinical experience in the screening and management of a large kindred with familial isolated pituitary adenoma due to an aryl hydrocarbon receptor interacting protein (AIP) mutation. J Clin Endocrinol Metab 99(4):1122-31.
